# The Anti-inflammatory Compound Candesartan Cilexetil Improves Neurological Outcomes in a Mouse Model of Neonatal Hypoxia

**DOI:** 10.3389/fimmu.2019.01752

**Published:** 2019-07-24

**Authors:** Sean Quinlan, Paula Merino-Serrais, Alessandra Di Grande, Heiko Dussmann, Jochen H. M. Prehn, Tríona Ní Chonghaile, David C. Henshall, Eva M. Jimenez-Mateos

**Affiliations:** ^1^Department of Physiology and Medical Physics, Royal College of Surgeons in Ireland, Dublin, Ireland; ^2^Division for Neurogeriatrics, Department of Neurobiology Care Sciences and Society, Center for Alzheimer Research, Karolinska Institutet, Stockholm, Sweden; ^3^Departamento de Neurobiologia Funcional y de Sistemas, Instituto Cajal, Consejo Superior de Investigaciones Cientificas, Madrid, Spain; ^4^FutureNeuro Research Centre, Royal College of Surgeons in Ireland, Dublin, Ireland; ^5^INFANT Research Centre, UCC, Cork, Ireland; ^6^Department of Physiology, School of Medicine, Trinity College Dublin, The University of Dublin, Dublin, Ireland

**Keywords:** neonatal encephalopathy, inflammation, microglia, seizures, hypoxia

## Abstract

Recent studies suggest that mild hypoxia-induced neonatal seizures can trigger an acute neuroinflammatory response leading to long-lasting changes in brain excitability along with associated cognitive and behavioral deficits. The cellular elements and signaling pathways underlying neuroinflammation in this setting remain incompletely understood but could yield novel therapeutic targets. Here we show that brief global hypoxia-induced neonatal seizures in mice result in transient cytokine production, a selective expansion of microglia and long-lasting changes to the neuronal structure of pyramidal neurons in the hippocampus. Treatment of neonatal mice after hypoxia-seizures with the novel anti-inflammatory compound candesartan cilexetil suppressed acute seizure-damage and mitigated later-life aggravated seizure responses and hippocampus-dependent learning deficits. Together, these findings improve our understanding of the effects of neonatal seizures and identify potentially novel treatments to protect against short and long-lasting harmful effects.

## Introduction

Hypoxic-ischemic encephalopathy (HIE) is the lead precipitator of neonatal seizures and a leading cause of long-term disabilities in infants (1). Survivors are at an increased risk of autism, epilepsy, cognitive and learning difficulties and psychiatric conditions (1, 2). Common clinical practice is to suppress neonatal seizures by using pharmacotherapies that boost inhibitory drive; such as phenobarbital. Response rates to phenobarbital are poor however, and treatment does not attenuate long-term neurological comorbidities (1–3). There are also concerns that phenobarbital and other anti-seizure medications may be neurotoxic at this age (4). The long-term benefits of non-pharmacological treatment such as therapeutic hypothermia are not yet established (1, 5). Therefore, there is an urgent need to identify novel mechanisms and treatments for neonatal brain damage, seizures and its long-term neurological outcomes.

Inflammation is increasingly implicated as a pathomechanism underlying neonatal brain damage following HIE (6–8). Early inflammation in the developing brain has been implicated in neurological disorders, including schizophrenia-like behavior and memory and attention disorders in experimental models (9–14). A hallmark of neuroinflammation is the induction and activation of glial cells; microglia and astrocytes, which produce soluble inflammatory cytokines such as interleukin 1β (IL1β) and tumor necrosis factor alpha (TNFα) (8). The hippocampus, key structure in memory and cognition brain function, is particularly enriched in cytokine receptors and primed to respond to inflammatory signaling (15, 16). Pre-clinical animal models of neonatal seizures have recently extended these insights, showing pharmacologic inhibition of IL1β production reduces neonatal seizures (17). Exposure of the neonatal brain to infection is also associated with lasting changes to the morphology of neurons within vulnerable brain structures (18), such as the hippocampus. Together, these findings suggest that early-life insults promote neuroinflammation that contributes to detrimental short- and long-term neurologic outcomes.

Microglia, which make up 6% of cells in the human brain (8) are dynamic cells that are critical for initiating and resolving inflammation in the brain (19–22). Along with their functions as an immune cells in the brain, microglia also perform neuronal pruning during a critical stage of brain development (8, 23–27). Accordingly, disruption of the CR3/C3 (complement receptor 3/complement derived activation fragment 3 pathway) specific microglia activation pathway during the postnatal period results in long-term deficits in synaptic connectivity (28).

Sartans are a family of small molecules that have recently emerged as novel anti-inflammatory drugs; this includes Candesartan cilexetil (CND). Anti-inflammatory effects of CND were originally attributed to blockade of the Angiotensin II receptor type 1 (AT1 receptor), but CND has also been shown to activate the anti-inflammatory nuclear receptor Peroxisome proliferator-activated receptor gamma (PPARγ) and, more recently, inhibit the microglia receptor Toll-like receptor 2 (TLR2) (29). CND recently entered clinical trials for the treatment of Parkinson's disease indicating potential use as a treatment for neuroinflammatory conditions (30–32).

Here, we hypothesize that hypoxic-seizures in the neonatal period activate inflammation and microglia, leading to pathological structural changes in the brain. Consequently, targeting inflammation may improve long term neurological outcomes after hypoxia-seizures. Here we extended previous insights into the pathophysiologic consequences of hypoxia-induced seizures in mice by assessing populations of glia, the time course of the inflammatory response and attendant structural changes of neuronal architecture in hippocampal neurons. We then show CND reduces seizure burden in a model of hypoxia-seizures when given as a pre-treatment, while post-treatment with CND reduces neuronal damage, normalized anxiety-like behavior and susceptibility to a second pro-convulsive agent in later life. Together, these results extend evidence that neonatal seizures cause lasting structural changes to the developing brain and support anti-inflammatory drugs as novel therapeutic strategies to attenuate the consequences of neonatal brain injury.

## Materials and Methods

### Mouse Model of Neonatal Hypoxia-Induced Seizures

All animal procedures were performed in accordance with the principles of European Communities Council Directive (86/609/EEC, 2010/63/EU), under license (REC#1203b) from the Department of Health and Health Products Regulatory Authority (Ireland) and procedures were approved by the Research Ethics Committee of the Royal College of Surgeons in Ireland. Neonatal litters of C57BL/6J mice [weight, 4–6 g; age, postnatal day 6.5–7.5 (P7)], were obtained from the Biomedical Research Facility, RCSI. Pups were kept with their dams in a barrier-controlled facility on a 12 h light–dark (7 a.m.−7 p.m.) standard cycle with access to food and water *ad libitum*. All experiments were performed during the light cycle.

Hypoxia in mouse pups was carried out as previously described (33). Male and female pups from the same litter were randomly placed in a clear hypoxic chamber and exposed to a premixed gas containing 5% O_2_/95% N_2_ for 15 min at 34°C and 80% humidity. Under these conditions, 97% of the pups develop seizures during and after hypoxia. Normoxic control pups were placed in the chamber at 21% oxygen (room-air) for the same period of time. All animals were observed during the 15 min of hypoxia.

For electrographic recording, mice were placed in a stereotaxic frame and anesthetized with isofurane/oxygen (5% for induction, 2–3% maintenance). Temperature was maintained with a heat-pad (Harvard Apparatus Ltd., U.K.). Isofurane exposure was limited to 8–10 min (2 min induction and maximum of 8 min during surgery), where three partial craniectomies were performed and tethered electrodes (stainless steel screws soldered to a Teflon-insulated stainless steel wire; E/363/20, Bilaney Ltd., U.K. Diameter: 0.56 mm) were fixed to approximately 1 mm depth while carefully avoiding the underlying brain surface and secured into the skull with dental cement. One electrode was placed in each temporal cortex (−5 mm AP and ± 2.5 mm ML from Bregma) and one reference in the cerebellum.

For analysis of EEG data, EEG was recorded using a Xltek digital EEG-amplifier and digitalized with Twin software using Notch filter (1 Hz high-pass and 60 Hz low-pass) (Grass Technologies Ltd., Warwick, RI). Then, filtered files were uploaded to LabChart Pro (V7, ADInstruments Ltd.). Seizures were defined as electrographic polyspike discharges ≥5 Hz, ≥ 2x baseline EEG amplitude and lasting ≥3 s. EEG total power [(μV2) is a function of EEG amplitude over time] was analyzed by integrating frequency bands from 0 to 60 Hz. For these analyses, hypoxia ictal and inter-ictal traces were selected and the values were normalized to the baseline of each animal (pre-hypoxia). The number and duration of seizures (measured as the time from first spike to last spike) were calculated per hypoxic episode of EEG recording. Total seizure burden was calculated as the accumulative time that pups or mice were having electrographic polyspikes discharges. Power spectral density heat maps were generated within LabChart (spectral view), with the frequency domain filtered from 0 to 80 Hz and the amplitude domain filtered from 0 to 40mV.

Behavioral/clinical seizures were scored using a modified 5 points Morrison's scale for scoring hypoxia/ischemia-induced seizures in neonatal mice: 0: Normal behavior; 1a: Immobility/motionless; 1b: Immobility and myoclonic jerks (spasms/shiver); 2: Rigid/ loss of posture; 3: Circling, swimming, peddling, tail extension; 4: Spasms, forelimb tonic-clonic; 5: Score 4 repeatedly.

### *In vivo* Administration of Pro- and Anti-inflammatory Drugs

**Figure 1:**
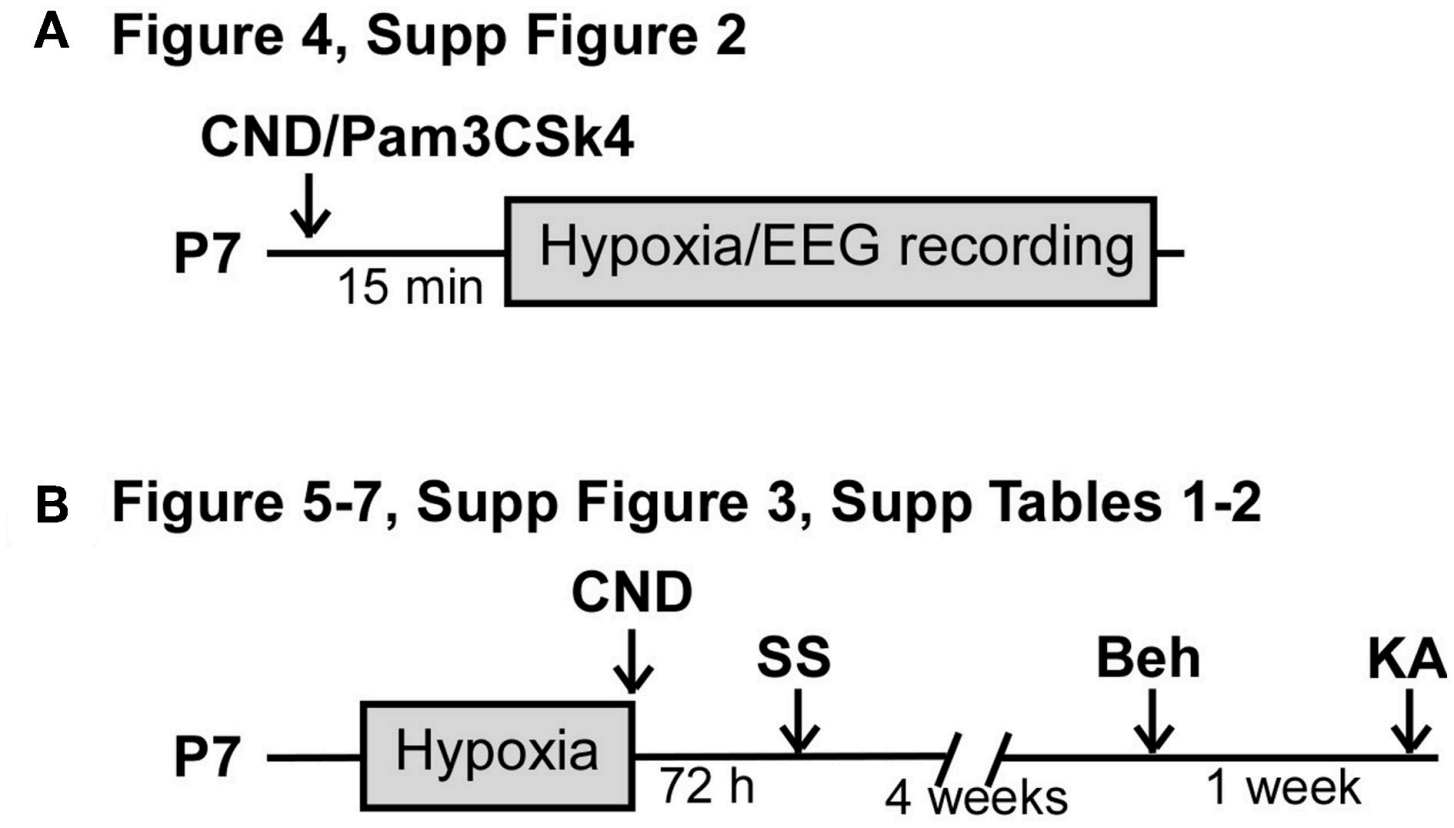
Experimental paradigm of drug administration. **(A)** Experimental paradigm for injection of CND or Pam3CSK4 pre-hypoxia induced seizures. Pups received a single dose of CND and Pam3CSK4 15 min before hypoxia. Electroencephalogram (EEG) was recorded during the 15 min of hypoxia. **(B)** Experimental paradigm for injection of CND post-hypoxia induced seizures. Pups were placed on the hypoxia chamber, and received a single dose of CND after hypoxia. Pups returned to the damn and analyzed for neuronal damage (72 h post-hypoxia), behavioral tasks (4 weeks post hypoxia) or KA susceptibility (5 weeks post-hypoxia).

To assess modulation of inflammation, P7 pups received an injection of vehicle or drug intraperitoneal [i.p., Candesartan Cilexetil (CND, Sigma): 1 mg.kg^−1^, vehicle: 1% DMSO in PBS (29, 34); Pam3CSK4 (Invivogen): 80 μg.kg^−1^, vehicle: PBS (35, 36)]. For the measurement of EEG and inflammatory markers experiments (Figures 1A), drugs were injected 15 min pre-hypoxia. For silver-staining and long-term studies, CND or vehicle was injected upon hypoxia cessation (Figures 1B). Pups prepared for EEG recordings were sacrificed immediately upon cessation of EEG. For long-term studies, pups were subjected to hypoxia without EEG electrodes and returned to the home cage.

### Cytokine Detection by Enzyme Linked Immunosorbant Assay (ELISA)

Hippocampi were extracted on ice and homogenized in lysis buffer (20 mM Tris HCl (pH: 7.2), 150 mM NaCl and 1% Triton and protease and phosphatase inhibitors [10 μg/ml Aprotinin (Sigma), 10 μg/ml Leupeptin (Sigma), 1 mM Vanadate (Sigma) and 1 mM PMSF (Sigma)], by dounce homogenizers. Cells debris and membranes were discarded by centrifugation at 10,000 rpm for 10 min and protein levels were quantified using Pierce BCA assay kit following manufacturer's instructions (ThermoFisher).

ELISA was performed as per manufacturer's instructions (RND Systems). Briefly, capture antibody was incubated at room temperature overnight, protein standard curve and 30–50 μg of protein samples were added in triplicate and incubated for 3 h at room temperature. Next, the detection antibody was added to each well and incubated at room temperature for 2 h. For visualization of the signal, a solution of streptavidin-HRP was added at a 1:40 dilution per well and a mix containing equal volumes of Color Reagents A and B. Finally, the enzymatic reaction was stopped and the plate was read immediately at 570 and 450 nm.

The cytokine concentration was calculated following manufacturer recommendation. Briefly, the 570 nm values were subtracted from the 450 nm values, standard curve values were plotted in GraphPad (Prism v7) and a line of best fit (second order polynomial) was generated. The amount of protein was extrapolated and the average of the triplicate was taken. The concentration was then normalized per mg of total protein in the tissue.

### Quantitative Real Time PCR

Hippocampi were isolated and total RNA was extracted using Trizol method as described previously (37). RNA concentration was determined using NanoDrop 2000, and RNA purity was measured by establishing the ratio of absorbance between 260 and 280 nm (260/280 ratio). One microgram of total RNA was retro-transcribed using SuperScript III Reverse Transcriptase.

Quantitative real-time PCR was performed using a LightCycler 1.5 (Roche Diagnostics) in combination with QuantiTect SYBR Green PCR kit (Qiagen) as per manufacturer's protocol. Specific primers were purchased from Sigma: *IL1*β (F: 5′ tgaagttgacggaccccaaa 3′, R: 5′ agcttctccacagccacaat 3′); *Tnf* α (F: 5′ ctcttcaagggacaaggctg 3′, R: 5′ cggactccgcaaagtctaag 3′); *Iba1* (F: 5′ tggaggggatcaacaagcaa 3′, R: 5′ accccaagtttctccagcat 3′); *Gfap* (F: 5′ agaaaaccgcatcaccattc 3′, R: 5′ tcacatcaccacgtccttgt 3′) and β*-Actin* (F 5′-ggttggccttagggttcagg-3′, R 5′-gggtgtgatggtgggaatgg-3′).

### Flow Cytometry-FACS

Prefrontal and temporal cortex and whole hippocampi were dissected from mice and processed for flow cytometry. Upon being removed from the skull the brain was stored in MACS tissue solution (Miltyeni). Tissue was dissociated using MACS Miltyeni Neural Dissociation kit (Papain) as per the manufacturer's instructions. Tissue was dissociated and filtered through 70 μm mesh filters (MACS). This cell mixture was then centrifuged at 1,200 rpm for 5 min and the cell pellet was suspended with FACS buffer (0.5 M EDTA, 1% FBS in PBS). 2.5 × 10^5^ cells were blocked with FcR Block solution (1 μL every 10^6^ cells) for 15 min on ice and then stained with anti-CD45-PE (1:100. eBiosciences. Thermo Fisher) and anti-CD11b-FITC (1:100. BioLegend) or appropriate control-IgG antibodies for 30 min on ice. Cells were then washed in FACS buffer and analyzed on FACS (BD FACSCANTO™ II). The data were analyzed with BD FACSDIVA™ software. The microglia subpopulation was defined as percentage of cells in the CD11b^+^/CD45^low^ gate (Supplementary Figure 1).

### Histopathology and Immunofluorescence

#### Bromo-Deoxy-Uridine (BrdU) Detection

BrdU was used to label dividing cells in the brain. Pups received a single injection of 50 mg.kg^−1^ of BrdU intraperitoneal 6 h pre-hypoxia. Seventy-two hours post hypoxic-seizures pups were perfused with cold PBS followed by 4% PFA. Brains were then embedded in agarose and cut at 50 μm using a Vibratome (VT1000). First, slices were incubated in 2 N hydrochloric acid (HCl) at 37°C for 30 min, then, sections were incubated in borate buffer for 15 min to neutralize any residual acid and prevent any further permeabilization. Sections were then washed in TBS for 5 min and rinsed in blocking solution (3% horse serum in 0.1% Triton X100-TBS). All sections were incubated with mouse anti-BrdU (1:400, Roche).

Slices were washed in TBS and incubated with rabbit anti-NeuN (1:500, Merck Millipore), rabbit anti-GFAP (1:500, Roche) or goat anti-Iba1 (1:500, Wako). Then, slices were washed, and incubated in secondary fluorescent antibodies, anti-mouse Alexa-488, anti-rabbit Alexa-568 anti-goat Alexa-568 (1:500, Invitrogen) in dark. Remaining antibody was washed with TBS and finally sections were incubated in Hoechst nuclear stain for 10 min. Sections were mounted onto slides and covered with FluorSave (Sigma). Brain slices (medial hippocampus) were visualized under the fluorescence microscope (Leica, DM4000B).

### Silver Staining

For histological analyses 72 h post hypoxia, pups were transcardially perfused with cold PBS followed by cold 4% PFA/2.5% glutaraldehyde in 0.1 M phosphate buffer, and post-fixed in the same solution. Brains were then fixed in 4% agarose and sectioned using a Vibratome at 50 μm (Leica VT1000). Then, sections were mounted onto superfrost slides (ThermoScientific) and dried overnight at 37°C and processed using a method developed by Gallyas et al. (38), with minor modifications (33). Briefly, after fixation slices were rinsed in 1% acetic acid, then sections were dehydrated with 1-isopropanol at increasing concentrations (50, 75, 97%). Finally, sections were esterified with 100% 1-propanol containing 0.8% sulfuric acid (Sigma) and incubated at 56°C for 18 h. Upon rehydration with decreasing concentration of 1-isopropanol and 1% acetic acid treatment, sections were developed in a mix of equal volumes of 10% Na_2_CO_3_ and 0.2% AgNO_3_, 0.25% NH_4_NO_3_, 2% tungstosilicic acid (Sigma) and 0.4% formaldehyde (Sigma). Sections were dehydrated, cleared with three rounds of histoclear and covered with DPX (Sigma-Aldrich). Semi-quantitative analysis of silver staining (medial hippocampus) was performed, by an experimenter blinded to the condition, under 20X lens magnification under the microscope (Leica DFC300FX). Two consecutive slides were counted per condition and the average number of positive cells from both slides was calculated.

### Intracellular Injection and Cell Reconstruction Analysis

Neuronal labeling technique was carried out as previously described (39). Mice exposed to normoxia or hypoxia 5 weeks earlier were used in the analysis. Pyramidal cells from the CA1 subfield (medial hippocampus) of 150 μm vibratome-prepared sections were individually injected with Lucifer Yellow by continuous current, filling individual dendrites until the distal tips of each cell fluoresced brightly, indicating that the dendrites were completely filled and ensuring that the fluorescence did not diminish at a distance from the soma. Sections were immunostained using rabbit polyclonal antibodies against Lucifer yellow produced at the Cajal Institute, followed by streptavidin coupled to Alexafluor 488 (1:1,000, Molecular Probes). From each mouse 3–6 neurons were randomly selected and the basal tree (stratum oriens) was scanned from the soma to the tip of the longer dendrite using LSM710 confocal laser scanning microscope (Carl Zeiss, ZEN 2009) with 20x 0.5NA (zoom 1.0), objective taking a z-stack with an interval of 1 μm between single images. To analyze the complexity of the dendritic tree, Z-stacks were combined in Image J and Neuron J plugin (40, 41) was used to manually draw the dendritic tree using the maximum intensity projection. All morphological analysis was performed blindly by the same investigator.

**Figure 2:**
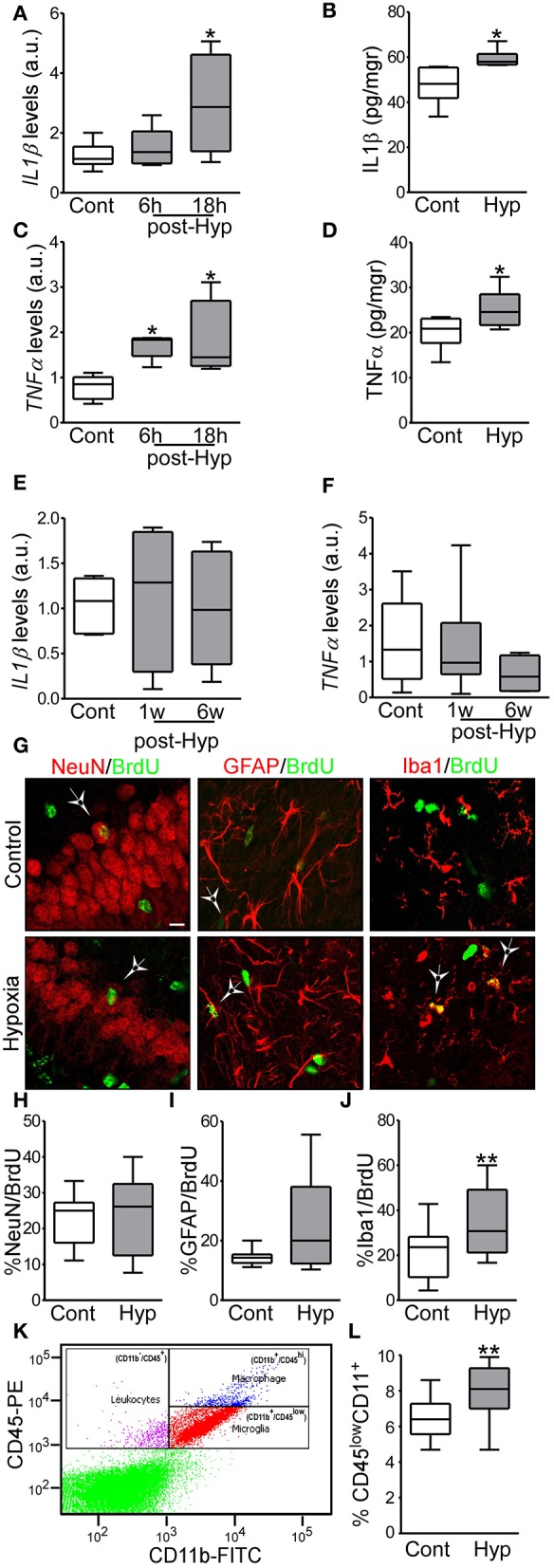
Hypoxia-induced seizures induce acute inflammation and microglia proliferation in a neonatal mouse model. **(A,B)** IL1β mRNA and protein levels are increased acutely in hypoxia-exposed pups compared to control [*n* = 6 per group. *p < 0.05. **A**, F: 4.854, t: 3.028 (vs. control), t: 0.568 (vs. 6 h). **B**, F: 4.477, t: 2.485)] **(C,D)** TNFα mRNA and protein levels are increased acutely in the hippocampus in hypoxia exposed mice compared to control [*n* = 6 per group. *p < 0.05. **C**, F: 6.736, t: 2.975 (vs. control), t: 2.138 (vs. 6 h). **D**, F: 1.322, t: 2.260)]. **(E,F)** Levels of IL1β and TNFα were normalized at 1 week and 6 weeks after hypoxia (*n* = 6 per group). **(G)** Representative confocal images of double-staining cells with BrdU and NeuN (neuronal marker, NeuN/BrdU) or GFAP (astrocytic marker, GFAP/BrdU) or Iba1 (microglia marker, Iba1/BrdU) in the hippocampus 72 h post hypoxia. Scale bar: 10 μm. **(H–J)** Quantification of the percentage of BrdU positive cells are also positive for NeuN **(H)**, GFAP **(I)** or Iba 1 **(J)** (*n* = 16 per group. **p < 0.01. **J**, F: 1.471, t: 3.133). **(K)** Representative scatter plot shows the distribution of CD45 and CD11 from control brain. **(L)** Quantitative analysis of the percentage of CD11^+^/CD45^low^ as a representation of the microglia population in control and hypoxia exposed pups (*n* = 6 per group. **p < 0.01. F: 2.397, t: 3.831).

For the overview image (Figure 2A), image was scanned with multiple fields of view of a 20x 0.8 objective, zoom 0.6, on a LSM 710 confocal microscope with an overlap of 10% of the FOV size using the multiple time series macro in ZEN 2009. Images were subsequently stitched using the stitching plugin (http://fly.mpi-cbg.de/~preibisch/contact.html) running in Image J (version 1.50i, Wayne Rasband, NIH).

### Cognitive and Behavioral Testing

#### Open-Field Test

Cognitive functions and behaviors were assessed in mice 4 weeks after hypoxia. To assess mobility, mice were studied in the open field test. The open-field test consists of an open glass box (30 × 30 × 20 cm) and was carried out prior to the novel object-location task (see below). Each mouse was placed in the center of the arena and allowed to explore for 10 min. Total distance traveled, velocity (cm.s^−1^), freezing episodes, number of crossings and average time spent in specific central/peripheral zones of the arena were quantitatively analyzed using video tracking (Ethovision, Tracksys, Nottingham, U.K.). The central and peripheral zones were designated post-testing using the Ethovision software.

#### Light-Dark Box Test

The test apparatus consisted of an open glass box (30 × 15 × 20 cm) connected to an acrylic dark box (30 × 15 × 20 cm) via an entrance. The light area was illuminated from above by a white lamp bulb (60 W/600 lux). Mice were habituated in the behavioral recording room 2 h prior to the beginning of testing. Mice were placed in the center of the light area, facing away from the entrance of the dark area, and were allowed to explore the apparatus for 10 min. Anxiety-related behavior was quantitatively assessed in terms of latency to enter the dark area by Ethovision tracking software.

#### Novel Object-Location Test

The test was carried out over two consecutive days in 10 min sessions. Day 1 (habituation day), mice were allowed to explore the arena without objects (10 min) followed by 3 sessions with two objects (10 min each session); on day 2 (test day), mice were placed in the same arena with one of the objects moved to a novel position. Object exploration was manually recorded and defined as the time mice were interacting with the objects with their nose or paw within 1 cm of the objects (3). To calculate the percentage of time with the novel-object, the following equation was used: (A)/(A+B)^*^100. A = Time spend with novel object. A+B = Time spend with both objects.

### Seizure Susceptibility Testing at Adulthood

A sub-group of pups subject to manipulations at P7 were later exposed to chemoconvulsant to assess seizure severity. For these studies, mice at 6 weeks of age received a single injection of kainic acid (15 mg.kg^−1^; KA, intraperitoneal). All mice receiving KA were recorded for both electrographic (EEG) and behavioral seizures. Electrode implantation, EEG recording and quantification were performed as previously described (3, 33). Briefly, mice were injected with KA and monitored for 60 min post-KA. During this time all clinical seizures were monitored in 5 min intervals. Behavioral scores were based on a 6-point Racine-like scale, as described previously (33). Racine's behavior scale: 0: normal behavior; 1: Immobility; 2: Freezing/tail extension; 3: Repetitive movements/ head bobbing; 4: Rearing and falling; 5: Tonic-clonic; 6: Repeated tonic-clonic. EEG was quantified as before.

### Statistical Analysis

All analyses were carried out by an observer blinds to the experimental condition. All data are presented as Box and Whiskers plots to show first and third quartiles, median and minimum and maximum of all the data. All statistical tests were carried out in the Graph-Pad Prism software. Two group experiments were analyzed using Mann Whitney U-test for nonparametric distributed data. Multi-group comparisons were made using one-way ANOVA with Bonferroni post-test. *P*-value of < 0.05 was considered significant.

## Results

### Hypoxia-Induced Seizures Produce an Acute Inflammatory Response

We have previously reported that hypoxia-seizures during the neonatal period induces neuroinflammatory signaling and long-lasting impairment of neurological function in mice (3, 33). Now, we evaluated the levels of pro-inflammatory cytokines post-hypoxia. Hippocampi from control and hypoxia-exposed pups were isolated and levels of the pro-inflammatory cytokines, IL1β and TNFα, were evaluated by qPCR and ELISA (Figures 2A–F). Interestingly, we observed an increase in both cytokines in the first 24 h after hypoxia-seizures (Figures 2A–D). This increase was transient and not observed in samples collected from mice 1 week and 6 weeks after hypoxia (Figures 2E,F). Thus, hypoxia-seizures induce short-lasting increases in inflammatory cytokines in this model.

### Hypoxia-Seizures Induces a Selective Increase in the Number of Microglia in the Neonatal Brain

We evaluated whether the acute increase in pro-inflammatory cytokines lead to changes to the major resident cell populations in the immature brain (32). Here, we first evaluated whether hypoxia-induced seizures result in altered neurogenesis or gliogenesis in the hippocampus. The cell division marker BrdU was injected pre-hypoxia and then tissue sections were collected 3 days later and co-labeled for markers of mature neurons (NeuN), astrocytes (GFAP) or microglia (Iba1).

No differences were observed in the percentage of NeuN^+^ or GFAP^+^ cells co-labeled with BrdU between hypoxia-seizure and control groups (Figures 2G–I). However, numbers of Iba1-BrdU double-positive cells were increased in the hypoxia-seizure group compared to control (Figures 2G,J). Thus, hypoxia-seizures appears to selectively expand the microglia population in this model, at this time following seizures.

To support these findings, we used Fluorescence-Activated cell sorting (FACS) analysis to quantify microglia cells obtained from brains of mice 72 h after hypoxia-seizures. Control pups had ~6,500 microglia per 1 × 10^5^ cells, which corresponds closely to the expected proportion of these cells [~6.5% of the total number cells (Figures 2K,L)] and is similar to assessments of neocortical microglia populations in the developing human brain (42, 43). Corroborating BrdU results, hypoxia-exposed pups had ~8,000 microglia per 1 × 10^5^ cells, which corresponded to ~8% of the total cell population (Figures 2K,L).

Together, this data showed that neonatal hypoxia-seizures cause an increase in the population of microglia in the developing brain.

### Hypoxia Induces Long-Lasting Changes in Neuronal Morphology in the CA1 Subfield of the Hippocampus

**Figure 3:**
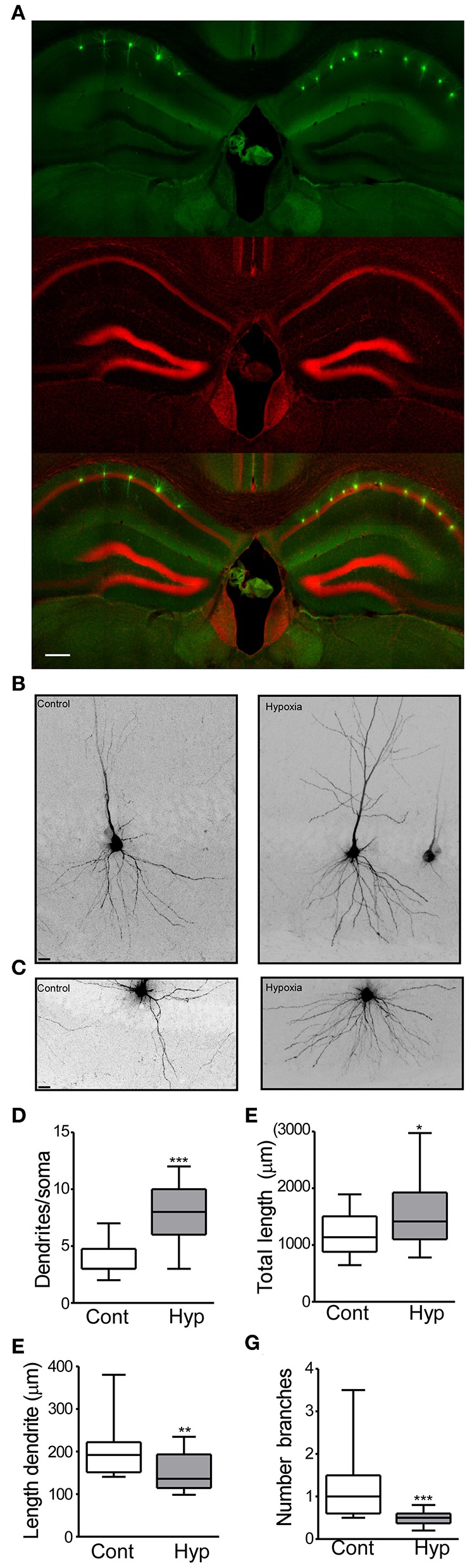
Hypoxia induces seizures at P7 results in neuronal morphology alterations later in life. **(A)** Field view of hippocampi showing Lucifer yellow injected CA1 neurons (green) and nuclei (Red). **(B)** Higher magnification CA1 injected neuron from the control (left) and hypoxia (right) group. **(C)** Representative images of the basal dendritic tree from the control and hypoxia group. **(D)** Number of basal dendrites per soma (F: 3.354, t: 6.854), **(E)** total dendritic length (F: 2.322, t: 2.435), **(F)** length of an individual dendrite (F: 1.588, t: 2.818) and **(G)** number of branches in control and hypoxia group (F: 16.90, t: 4.135). Scale bar: **(A)** 500 μm and **(B,C)** 10 μm. (*n* = 17–20 neurons per group from 4 mice per condition). (*P < 0.05, **p < 0.01, ***p < 0.001).

It has now emerged that a major function of microglia is early dendritic pruning (25). We hypothesized that the increase in neuro-inflammation and brain resident microglia would be associated with long-lasting alteration of the structure of pyramidal neurons, principle excitatory neurons. To test this idea, P7 pups were subjected to hypoxia-seizures and then 5 weeks later (at 6 weeks old) brain slices were obtained. CA1 pyramidal neurons were individually injected with Lucifer yellow. The complexity of the basal dendritic tree (*stratum oriens*) was analyzed by confocal microscopy (Figures 3A–C).

In the control group, 20 neurons (from 4 mice) and a total of 23,706 μm dendritic length were analyzed. On average, a neuron from the control group showed 3.7 dendrites (primary branches coming from the soma) (Figure 3D) with a total dendritic length of 1,185 μm (Figure 3E). Analysis of individual dendrites from the control group showed that a single dendrite was on average 197.3 μm long (Figure 3F) and one dendrite presented ~1.1 branches (Figure 3G). In contrast, CA1 pyramidal neurons from mice previously subject to hypoxia-seizures showed on average 7.9 dendrites (primary branches, Figure 3D) and a total dendritic length of 1,575 μm (Figure 3E). Our results correspond to an increase of 30% of the basal dendritic extension in mice previously subject to hypoxia-seizures compared to the control group. Additionally, individual dendrites were 150.3 μm in length, 25% shorter than a dendrite from the control group (Figure 3F) and presented 0.4 branches (Figure 3G). These findings indicated that neonatal hypoxia-induced seizures induce lasting and substantial morphological alterations of principal excitatory neurons in the *stratum oriens* from mouse hippocampus.

### Anti-inflammatory Drug CND Reduces Seizure Severity Following Neonatal Hypoxia

**Figure 4:**
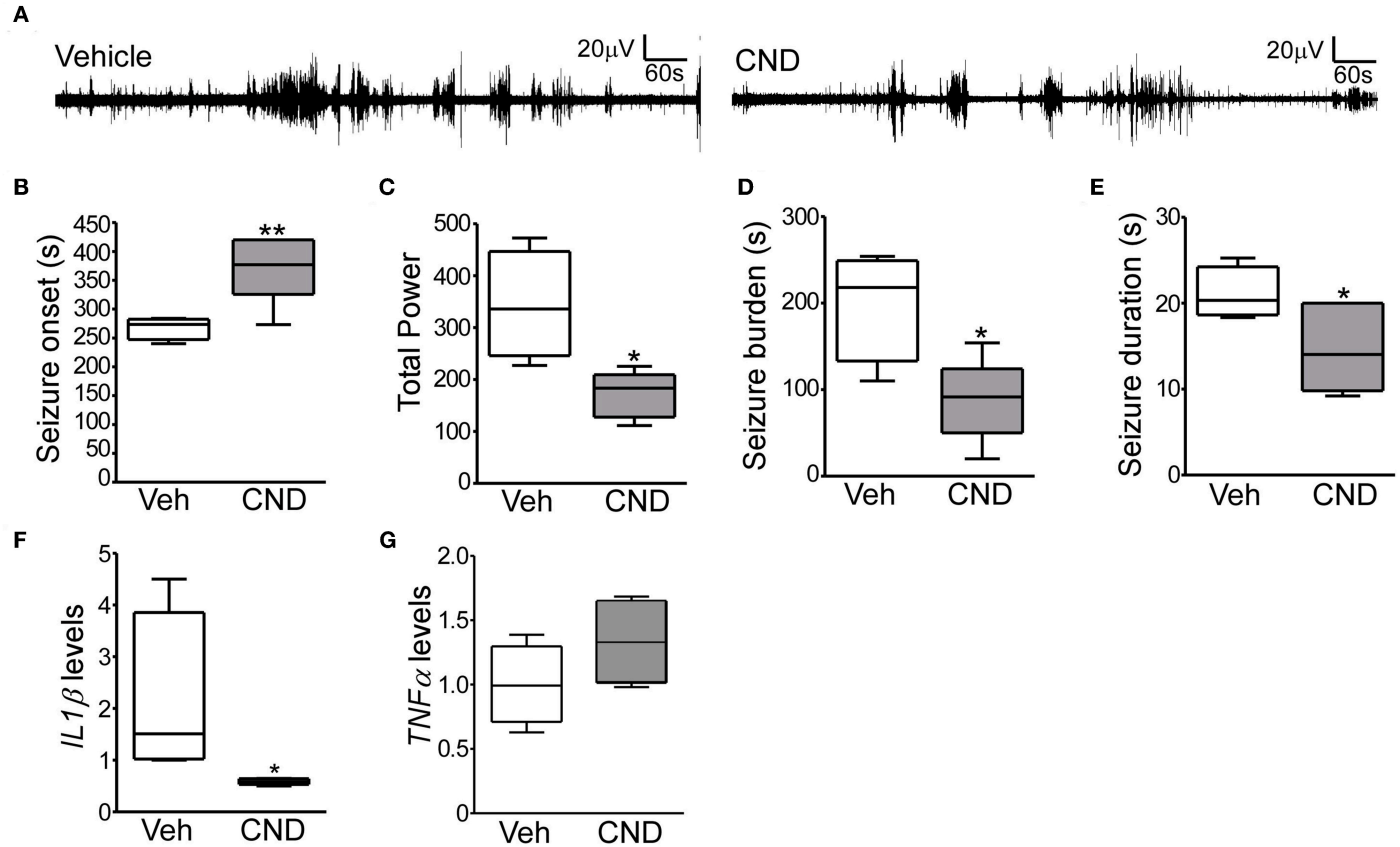
Targeting inflammation modulates hypoxia-induced seizures in P7 mice **(A)** Representative EEG traces from mice pre-treated with vehicle or CND and exposed to hypoxia-induced seizures. **(B–D)** Quantification of EEG files show that mice treated with CND have a delayed onset to first seizures (**B**; F: 8.309, t: 3.386), further, these mice also have significantly reduced in total power (**C**; F: 5.486, t: 3.361) and seizure burden (**D**; F: 1.913, t: 3.230) (*n* = 5–7 per group. *p < 0.05, **p < 0.01). **(E)** CND-treated pups experienced also significantly shorter seizures (*n* = 5–7 per group. *p < 0.05. F: 2.466, t: 2.411). Mice exposed to CND have a reduction in IL1β (**F;** F: 778.6, t: 2.085), without affecting TNFα **(G)** (*n* = 5 per group. *p < 0.05).

Given the inflammatory response induced by neonatal hypoxic-seizures and evidence that inflammation is a key patho-mechanism after hypoxia-induced seizures, we hypothesized that targeting inflammation would reduce seizures in the P7 model. For this, we selected the anti-inflammatory drug CND. CND was administrated 15 min before hypoxia, and electroencephalogram (EEG) was recorded and quantified (Figures 1A, 4A). CND injected mice showed a delay in seizure onset, a reduction in total power, seizure burden and duration of a single seizure compared to the vehicle injected pups (Figures 4B–E). As expected, levels of IL1β measured 24 h later were reduced in the CND-injected pups compared to vehicle injected pups (Figure 4F) and no changes were observed on TNFα (Figure 4G).

We next hypothesized that enhancing inflammatory responses should produce the opposite effect in the model. To test this idea, mice were pre-treated with the pro-inflammatory and microglia activating drug, Pam3CSK4 (44). As expected, total power, seizure burden and inflammatory markers were significantly higher in the PAM-injected mice compared to vehicle (Supplementary Figure 2), suggesting that modulating inflammation is sufficient to alter seizure severity in neonates.

### CND Reduces the Neuronal Damage Induced by Hypoxia-Seizures in Mice

**Figure 5:**
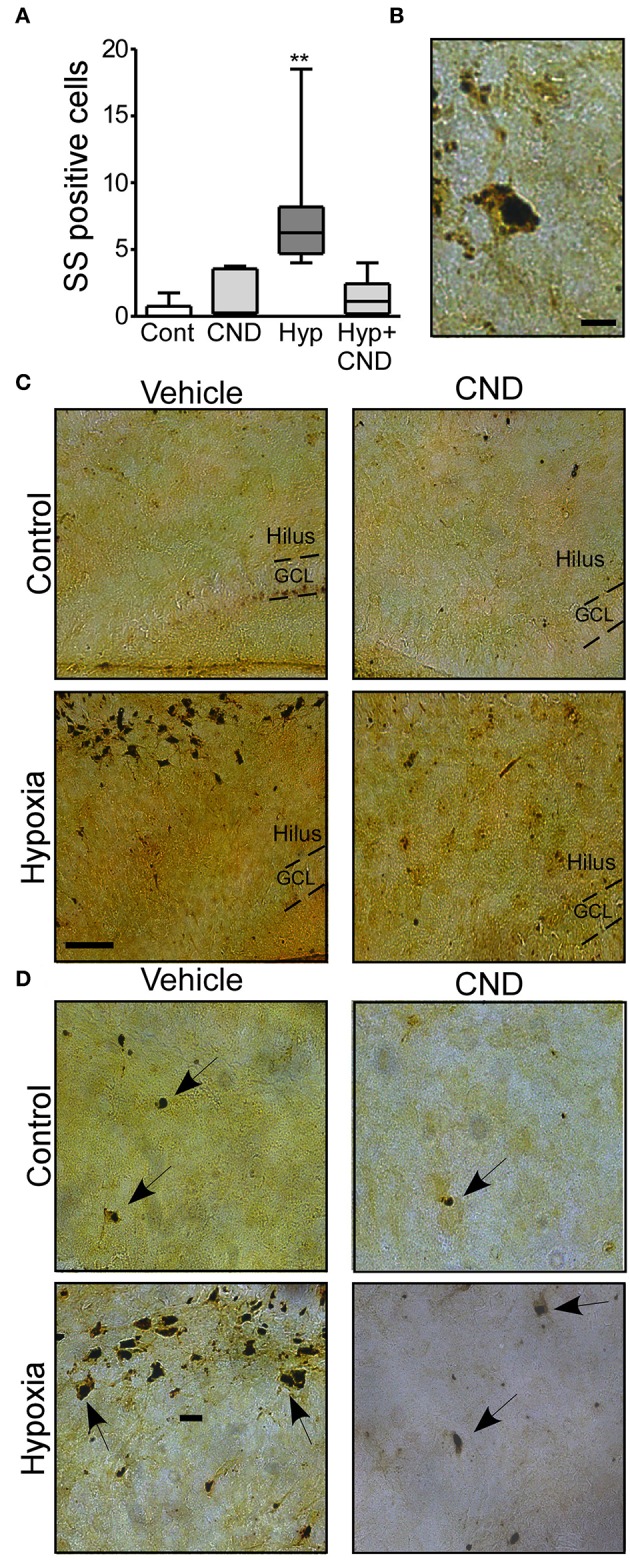
CND protects neonates against the neuronal damage induced by hypoxia. P7 pups were exposed to normoxia or hypoxia and receive a single dose of vehicle (veh) or CND just after hypoxia. **(A)** Graph shows the number of silver-staining positive cells in the four experimental paradigms. Note: CND protects against hypoxia induced neuronal damage [*n* = 6–8, **p < 0.01, compared to control. F: 11.500, t: 5.141 (vs. control), t: 4.308 (vs. CND), t: 4.553 (vs. Hyp-CND)] **(B)** Representative high magnitude (40x) silver staining images from the hypoxia group. Scale bar: 10 μm. **(C)** Representative 10x silver staining images of the hilus 72 h post-treatment. Scale bar: 50 μm. **(D)** Representative 20x images of the hilus area of control, CND and/or hypoxia exposed mice. Scale bar: 20 μm.

Next, we sought to determine if post-treatment with CND, a more clinically-relevant scenario would alter outcomes. Pups were subjected to normoxia or hypoxia and immediately after received a single dose of vehicle or CND. As previously described (3, 33), sporadic silver (SS) positive cells were observed in the control group (Figures 5A–D). Pups subject to hypoxia-seizures had significantly increased SS-positive neurons in the hippocampus (Figures 5A–D). Treatment of mice with CND alone or in combination with hypoxia was associated with only occasional silver positive cells similar to numbers in non-hypoxic controls (Figures 5A–D). These data suggested that post-treatment with CND protected against hypoxic-seizure neuronal damage.

### Effects of CND and/or Hypoxia-Induced Seizures on Locomotor Activity Later in Life

Previously, we have reported that hypoxia-seizures at P7 induced anxiety-like behavior, memory impairment and increases seizure susceptibility later in life (3). Now, we speculated whether post-treatment with CND may normalize the neurological outcomes associated with neonatal hypoxia-seizures (3).

**Figure 6:**
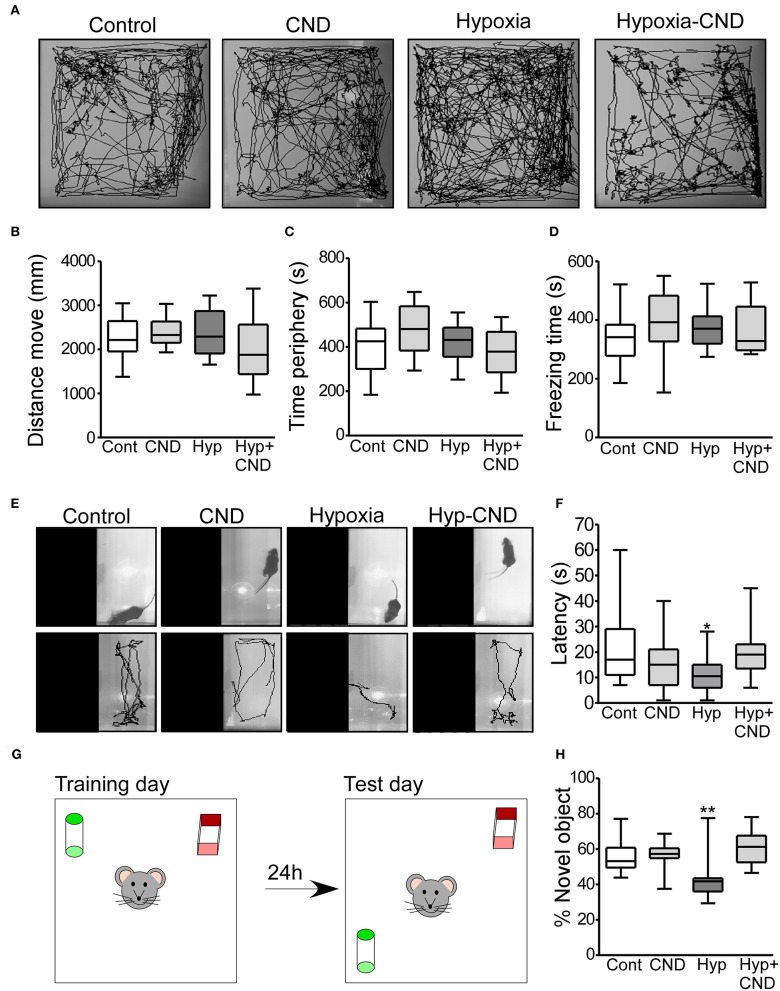
CND normalizes the anxiety-like behaviors and memory impairment caused by hypoxia-induced seizures. **(A–D)** Motor and anxiety-related behavior in mice exposed to hypoxia and/or receiving CND at P7. **(A)** Representative behavior tracking of controls, CND, hypoxia and hypoxia-CND in the open field test. **(B–D)** Distance move, time spent in periphery and freezing time in the 10 min of recording (*n* = 11–13). **(E,F)** Representative view above the light-dark box shows the location of the mouse at the start of the test and the tracks and graph quantifying the latency to the first transition to the dark zone [*n* = 11–13, *p < 0.05, **p < 0.01 compared to control, CND and Hyp-CND group. F: 4.036, t: 3.127 (vs. control), t: 1.723 (vs. CND), t: 2.918 (vs. Hyp-CND)]. **(G,H)** Schematic and quantification of the novel-object position test [*n* = 11–13, *p < 0.05, **p < 0.01 compared to control, CND and Hyp-CND group. F: 7.515, t: 3.126 (vs. control), t: 3.392 (vs. CND), t: 4.565 (vs. Hyp-CND)].

Separate groups of mice were injected with CND just after hypoxia at P7 and returned to the home-cage for 4 weeks and assessed at 5 weeks of age. First, the four different experimental groups (Control: Con; CND; Hypoxia: Hyp; Hypoxia-CND: Hyp-CND) were evaluated in an open arena (open-field task), and recorded for 10 min (Figures 6A–D).

Locomotor behaviors were analyzed over the complete 10 min recording. There were no differences in the parameters analyzed including the distance moved, time spent in the periphery and freezing time (Figures 6A–D). Thus, CND alone or in combination with hypoxia did not affect locomotor activity.

### CND Improves Anxiety-Like Behavior Induced by Hypoxia-Induced Seizures

As an assessment of anxiety-like behavior, mice were tested in the light-dark box task, with latency to enter the dark area used as an index of anxiety-like behavior (Figures 6E,F) (45). Control mice explored the light area for approximately 10–30 s before entering the dark area (Figures 6E,F). Similar behavior was observed in the normoxic pups that received CND. In contrast, and as reported previously (3), hypoxia-exposed mice moved directly into the dark area following release into arena (Figures 6E,F). However, the pups which received a single dose of CND after hypoxia at P7 explored the light area for 10–30 s, similar to the control group (Figures 6E,F).

### CND Protects Against Memory Dysfunction Caused by Hypoxia-Induced Seizures

We also assessed Mouse performance in the novel object-location task, a test of hippocampal function (46, 47) (Figures 6G,H). First, the total time spent with both objects was assessed. All experimental groups generally spent equal amounts of time interacting with both objects (Con: 22.7 ± 3.1 s; CND: 23.8 ± 2.3 s; Hyp: 21.7 ± 4.4 s and Hyp-CND: 20.2 ± 2.9 s).

When the percentage of time with the novel object-location was evaluated, the control group spent ~60% of the time with the object at the novel location (Figures 6G,H). As previously observed (3), the hypoxia-exposed pups spent 42% of the time interacting with the novel-location object (Figures 6G,H). However, the pups which received CND alone or after hypoxia explored the novel location for ~60% of the time, similar to the control group. When males and females were compared, no differences between sex were observed (Supplementary Table 1). This data showed that CND protected against the impairment of memory function caused by hypoxia-induced seizures.

### CND Reduces the Susceptibility to KA-Induced Seizures in Mice Exposed to Hypoxia-Induced Seizures During the Neonatal Period

**Figure 7:**
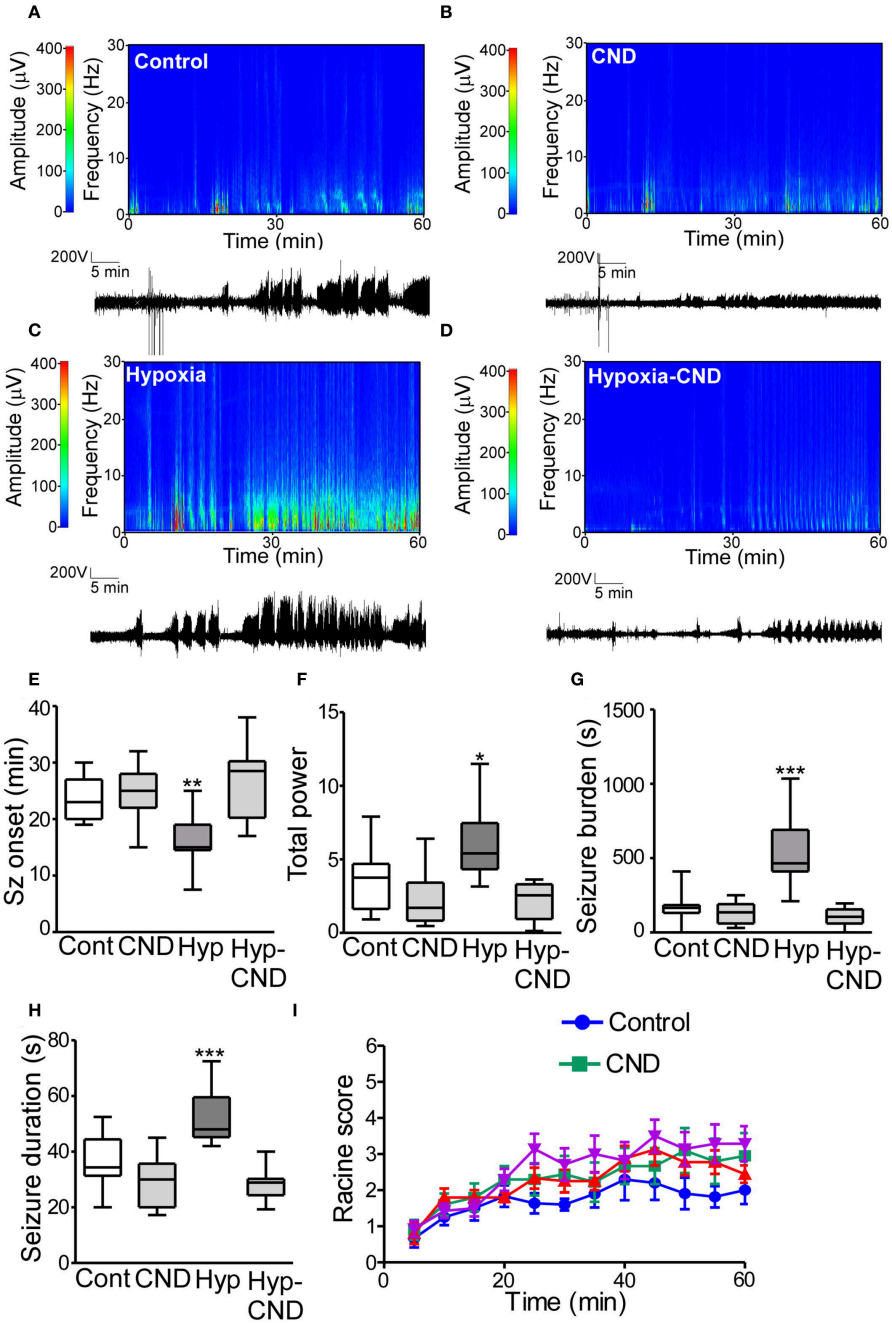
CND improves the susceptibility to develop seizures later in life after hypoxia-induced seizures in a neonatal mouse model. Five weeks after neonatal treatment, mice were implanted with electrodes and challenged with systemic KA (15 mg.kg^−1^), EEG and behavior were recorded. **(A–D)** Representative spectrograms and corresponding EEG traces from the control **(A)**, CND **(B)**, Hypoxia **(C)**, and hypoxia-CND group **(D)**. **(E–H)** Quantification of seizure onset, total power, total seizure burden, and seizure duration during the 60 min of recording. **(E)** Seizure onset was earlier in the hypoxia group compared to the control, CND and Hyp-CND group (*n* = 11–13, **p < 0.01 compared to control, CND and Hyp-CND group. F: 11.41, t: 3.896 (vs. control), t: 4.247 (vs. CND), t: 5.387 (vs. Hyp-CND)]. **(F)** Total power was higher in the hypoxia group compared to the control, CND and Hyp-CND group [*n* = 11–13, *p < 0.05 compared to control, CND and Hyp-CND group. F: 10.85, t: 3.183 (vs. control), t: 4.713 (vs. CND), t: 5.051 (vs. Hyp-CND)]. **(G)** Similar results were found in seizure burden in the hypoxia group [*n* = 11–13, ***p < 0.001 compared to control, CND and Hyp-CND group. F: 25.89, t: 6.514 (vs. control), t: 7.038 (vs. CND), t: 7.585 (vs. Hyp-CND)]. **(H)** Hypoxia-exposed pups developed on average longer seizures [*n* = 11–13, ***p < 0.001 compared to control, CND and Hyp-CND group. F: 18.000, t: 4.555 (vs. control), t: 6.050 (vs. CND), t: 6.381 (vs. Hyp-CND)]. **(I)** Seizure behavioral score taken every 5 min over the 60 min period using the modified Racine scale for mice (*n* = 11–13).

There is a long-standing association between early-life neonatal seizures and later life epilepsy. To test whether CND alters later-life seizure susceptibility we challenged the four different groups of mice (Con, CND, Hyp, and Hyp-CND) at 6 weeks of age with 15 mg.kg^−1^ i.p. of the chemoconvulsant KA. EEG and behavior were video-recorded for 60 min after KA injection (Figure 7).

EEG analyses showed that control mice developed typical epileptiform discharges 20–30 min post-KA injection (Figures 7A–E). In contrast, mice subject to neonatal hypoxia-seizures displayed a shorter time to seizure onset after KA challenge compared to the other groups (Figures 7A–E). Pups that received CND alone or CND after hypoxia developed similar seizure-like activity to controls after KA injection (Figures 7A–E). Quantitative analysis of the EEG supported these findings, when power and seizure burden were evaluated, we observed similar results. Hypoxia-seizure mice had higher EEG power and seizure burden after KA compared to controls, whereas CND and hypoxia-seizure mice given CND had a similar response to KA as the control group (Figures 7F,G). Similar results were observed when the duration of an individual seizure was evaluated with hypoxia-seizure exposed mice experiencing longer seizures compared to the control and CND and hypoxia-seizure CND groups (Figure 7H). When males and females were compared, no differences between sex were observed (Supplementary Table 2). No differences in clinical seizure behavior were observed between groups during the recordings (Figure 6I).

## Discussion

In the current work, we present evidence that hypoxia-induced seizures activate neuro-inflammation and increase microgliosis in the developing mouse brain. Hypoxia-seizures provoked upregulation of inflammatory cytokines and aberrant neuronal morphology in pyramidal neurons from the hippocampus. We show that a currently FDA-approved drug, the anti-inflammatory compound CND, not only suppresses acute hypoxia-induced seizures but also, reduces later-life anxiety-like behavior, seizure susceptibility in mice and preserves memory function. These results identify a novel experimental therapy that may attenuate long-term comorbidities.

Our study provides evidence that hypoxia-induced seizures increase microgliogenesis and elicit an acute inflammatory response, typified by an acute increase in IL1β. It has recently emerged that microglia perform critical functions during brain development including dendritic pruning (8, 23–26). An important finding in the present study was that pyramidal neurons in the CA1 subfield of mice subjected to hypoxia-induced seizures in the neonatal period developed shorter dendrites with fewer branches. This would be consistent with increased neuro-inflammation, and, perhaps due to the increased number of microglia (48). Notably, perinatal activation of inflammation by the viral mimetic PolyI:C, in rats reduces neuronal complexity in DG neurons in adulthood, and animals later developed schizophrenia-like behavior (49, 50). Morphological analysis of hippocampal neurons from patients with autism and schizophrenia also found shorter dendrites with reduced dendritic branching in pyramidal neurons (51), suggesting there is a casual link between neuronal morphology and behavioral outcomes. Accordingly, we speculate that independent of the initiating inflammatory response, sterile or infectious, inflammation caused by hypoxia-seizures leads to microglial activation and thus aberrant neuronal morphology, and ultimately enhancing the propensity for neurological disorders. Indeed, slight changes in the morphology of the dendritic tree of pyramidal neurons, particularly the dendritic length, alters the neuronal activity and ultimately cognition (52).

In this study, we have focused on the hippocampus due to its vulnerability to inflammation. The hippocampus presents the highest density of cytokines receptors compared to other areas of the brain, making it especially vulnerable to an inflammatory response (15, 16). However, we cannot discard the effects of hypoxia-seizures on other areas of the brain which may provoke a similar inflammation response along with morphological changes and ultimately contribute to the same neurological outcomes.

In the present study we observed that CND, a drug approved by the FDA and in clinical trials for Parkinson's diseases (31, 32), improves acute and long term outcomes following hypoxia-seizures in mice. Specifically, we observed that mice pre-treated with CND experience milder seizures during neonatal hypoxia. This is consistent with previous studies showing inhibitors of neuroinflammation protect against neonatal seizures (17). More importantly, post-treatment of pups subject to hypoxia-seizures reduced neuronal damage and mice performed better in hippocampal function-dependent tasks at adulthood. Taken together, these data suggest that targeting inflammation in the neonatal period may have both anti-seizure actions as well as protecting against the long-term consequences of hypoxia-induced seizures. Further studies would be required to elucidate whether CND elicits direct anti-seizure effects, or is protecting through a blunting of the inflammatory response, which has been shown to “prime” the brain for hyper-excitability (53, 54).

In our current study, we did not observe sex differences between males and females in the novel object location task or in the seizure susceptibility test, e.g., seizure onset, total power, seizure burden. We do acknowledge that the present model in mice may not capture the full spectrum of sex differences reported clinically in babies. Sex differences have been seen in humans in the response to neonatal brain damage, with boys developing worse outcomes compared to girls suffering from neonatal brain damage at birth (55, 56). Nevertheless, it is interesting to consider that similar outcomes might be produced by different mechanisms of action in males and females mice. The developing male brain is in a higher state of excitation and has longer periods of GABA-directed excitatory responses than females, while, microglia activation may have a favorable effect on females and not in males (57).

Neuroinflammatory signaling is an essential component in responding to injury and studies show that inhibition can also produce deleterious effects (58). Given this critical developmental stage in both neuronal maturation and immune function development, it would be pertinent to suggest that inhibiting the immune response at this time would also produce negative long-term outcomes. However, we did not observe any negative outcomes in those mice treated only with CND, suggesting that it is relatively safe to give in the neonatal period. Indeed, there have been several random, double-blinded control trials on the safety and tolerability of CND in adults, which have confirmed its neuroprotective properties and safety profile in Parkinson's disease (31). There is certainly a need for a safe and effective therapy for neonatal seizures. Current treatments are ineffective in about half of cases and there is concern anti-seizure drugs may induce negative neurological outcomes (3, 59–61). Notably, phenobarbital-treated children present with lower IQ ratio than un-treated children (59, 61). Likewise, a clinical trial of bumetanide, which may enhance inhibition in the brain, was halted due to a high occurrence of hearing loss in infants (1, 62). The efficacy of non-pharmacologic treatments, such as therapeutic hypothermia, is also uncertain with treated infants displaying lower IQ scores at follow-up and no improvement in mortality (1).

A number of questions remain to be answered: 1. Would we observe a similar response to CND in other models? For example, a premature model of neonatal encephalopathy or a model of more severe hypoxia. Since rodent models of encephalopathy in premature infants also feature inflammation and activation of microglia we can speculate that CND and anti-inflammatory drugs may have a similar protective effect (63). Since hypoxia-induced seizures in our model are insufficient to cause neuronal death we do not know if CND can protect against cell loss. This could be tested, for example in models with active neuronal death, such as the hypoxia-ischemia model in rats (64). Nevertheless, CND reduces the neuronal injury observed by silver staining. 2. It would be important to compare how CND performs relative to an approved anti-inflammatory drug. Would we observe similar effect using common used anti-inflammatory drugs in the clinic? Future extensive side-by side studies will be necessary to address this question. Nevertheless, non-steroidal anti-inflammatory drugs (NSAIDs) such as naproxen or ibuprofen do not affect activity evoked due to hypoxia *in vitro* (65), suggesting that the effect observed in the present study may be specific to CND.

In conclusion, we observe an increase in inflammation and changes in neuronal morphology after hypoxia-seizures in neonates. Targeting inflammation may protect against neonatal brain damage, reducing seizures during hypoxia, reducing neuronal damage afterwards and improving later-life neurological comorbidities, including anxiety-like behavior, hippocampal function and susceptibility to seizures. Here, we show the therapeutic potential of targeting inflammation as a novel treatment for the long-term outcomes after neonatal brain damage.

## Data Availability

The raw data supporting the conclusions of this manuscript will be made available by the authors, without undue reservation, to any qualified researcher.

## Ethics Statement

All animal procedures were performed in accordance with the principles of European Communities Council Directive (86/609/EEC, 2010/63/EU), under license (REC#1203b) from the Department of Health and Health Products Regulatory Authority (Ireland) and procedures were approved by the Research Ethics Committee of the Royal College of Surgeons in Ireland.

## Author Contributions

SQ performed the experiments. PM-S and EJ-M performed cell reconstruction analysis. HD supported with confocal images. AD and TN supported FACS experiments and analysis. JP and DH commented and edited the manuscript. SQ and EJ-M conceived, designed and wrote the manuscript. All authors read and approved the final manuscript.

### Conflict of Interest Statement

The authors declare that the research was conducted in the absence of any commercial or financial relationships that could be construed as a potential conflict of interest.
