# Supplementary material for: The Anti-inflammatory Compound Candesartan Cilexetil Improves Neurological Outcomes in a Mouse Model of Neonatal Hypoxia
Source: Front Immunol. 2019 Jul 24;10:1752. doi: 10.3389/fimmu.2019.01752 (PMC6667988; doi:10.3389/fimmu.2019.01752)
Supplement: Supplementary file 1 [file Table_1.DOCX]

|  | **Control** | | **CND** | | **Hypoxia** | | **Hypoxia-CND** | |
| --- | --- | --- | --- | --- | --- | --- | --- | --- |
|  | Male | Female | Male | Female | Male | Female | Male | Female |
| **%** | 62 | 38 | 53 | 47 | 53 | 47 | 30 | 70 |
| **Mean + SEM** | 56.63+3.11 | 51.66+  1.91 | 55.97+1.35 | 58.58+  5.64 | 39.04+2.48 | 46.71+  8.06 | 62.95+5.46 | 61.23+  3.61 |
| **P value** | 0.468 | | 0.6343 | | 0.350 | | 0.790 | |
| **F; t** | F=8.788;  t=0.838 | | F=14.40;  t=0.492 | | F=8.810; t=0.986 | | F=1.144;  t=0.269 | |

*Supplementary Table 1*: Effects of hypoxia-induced seizures and/or CND on the novel object-location task in males and females. No differences were found in any of the analysed parameters between males and females. Abbreviations: CND: Candesartan Cilexetil.
