# Supplementary material for: The Anti-inflammatory Compound Candesartan Cilexetil Improves Neurological Outcomes in a Mouse Model of Neonatal Hypoxia
Source: Front Immunol. 2019 Jul 24;10:1752. doi: 10.3389/fimmu.2019.01752 (PMC6667988; doi:10.3389/fimmu.2019.01752)
Supplement: Supplementary file 2 [file Table_2.DOCX]

|  | **Control** | | | **CND** | | | **Hypoxia** | | | **Hypoxia-CND** | | |
| --- | --- | --- | --- | --- | --- | --- | --- | --- | --- | --- | --- | --- |
|  | *Male* | *Femal* | *P value; F; t* | *Male* | *Femal* | *P value; F; t* | *Male* | *Femal* | *P value; F; t* | *Male* | *Femal* | *P value; F; t* |
| **Sz onset**  **(Mean+SEM)** | 22.63+ 1.43 | 45.00+ 22.55 | 0.112; 92.13; 1.762 | 23.50+1.98 | 24.33+1.20 | 0.789; 5.423; 0.278 | 17.67+1.93 | 13.75+1.28 | 0.121; 2.278; 1.695 | 23.00+3.61 | 28.33+0.33 | 0.215; 117.0; 1.473 |
| **Sz burden**  **(Mean+SEM)** | 158.8+11.37 | 360.0+275.0 | 0.222; 219.4; 1.314 | 121.7+19.69 | 140.0+63.51 | 0.729; 5.201; 0.362 | 470.8+110.3 | 635.8+95.15 | 0.284; 1.344; 1.133 | 108.3+30.87 | 155.0+32.79 | 0.359; 1.128; 1.036 |
| **Sz Duration**  **(Mean+SEM)** | 34.91+3.26 | 28.67+14.89 | 0.544; 7.791; 0.630 | 30.47+3.31 | 28.57+4.59 | 0.749; 1.040; 0.333 | 51.04+4.52 | 52.78+4.13 | 0.782; 1.199; 0.847 | 32.00+4.36 | 26.78+2.23 | 0.347; 3.821; 1.061 |

*Supplementary Table 2:* Effects of hypoxia-induced seizures and/or CND on seizure susceptibility in males and females. No differences were found in any of the analysed parameters between males and females. Note: Percentage of males and females are described on Supplementary Table 1. Abbreviations: Sz: Seizure. CND: Candesartan Cilexetil. Femal: Female.
