# Supplementary figures and images for: The Anti-inflammatory Compound Candesartan Cilexetil Improves Neurological Outcomes in a Mouse Model of Neonatal Hypoxia
Source: Front Immunol. 2019 Jul 24;10:1752. doi: 10.3389/fimmu.2019.01752 (PMC6667988; doi:10.3389/fimmu.2019.01752)

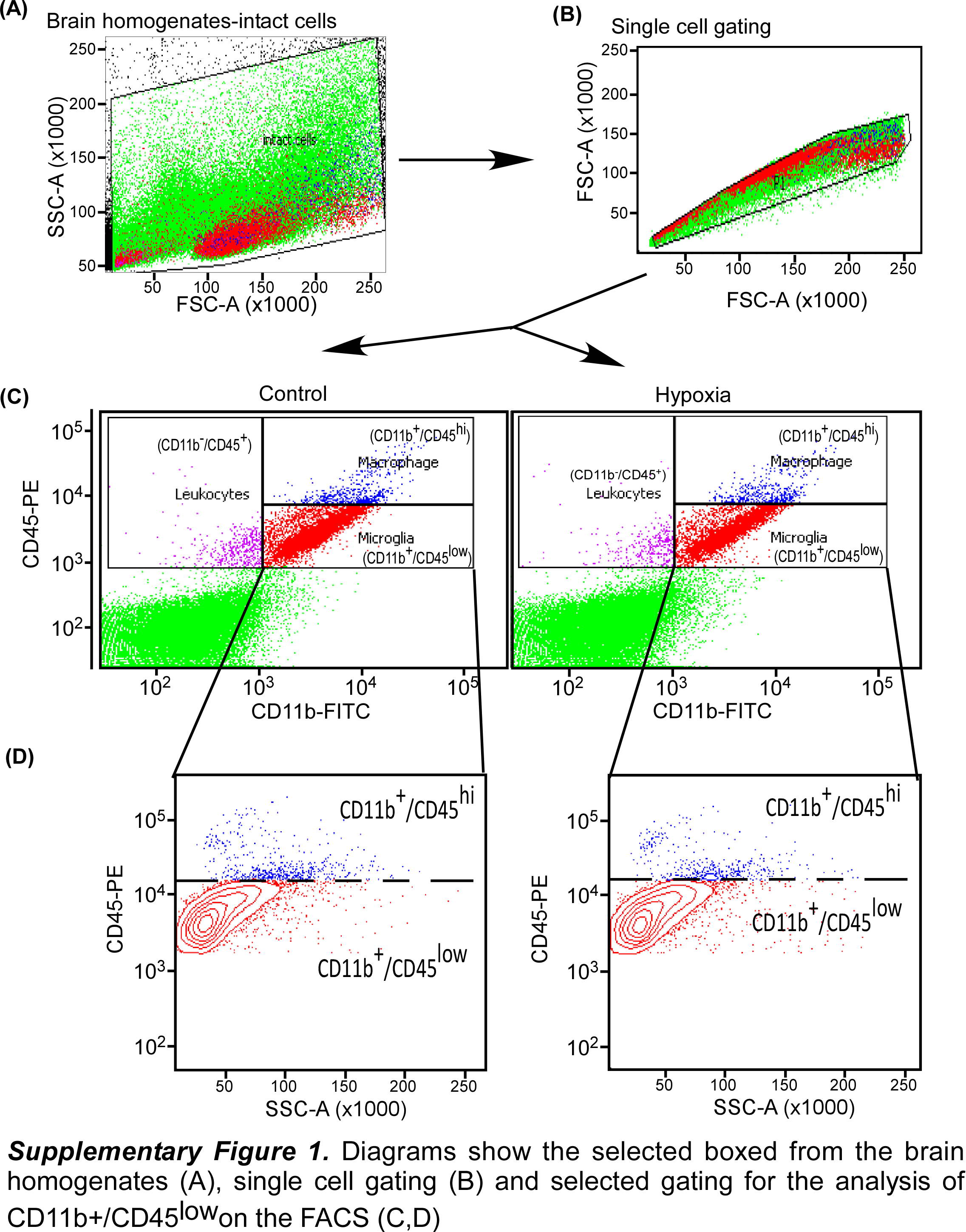

Supplement: Supplementary file 3 [file Image_1.JPEG]

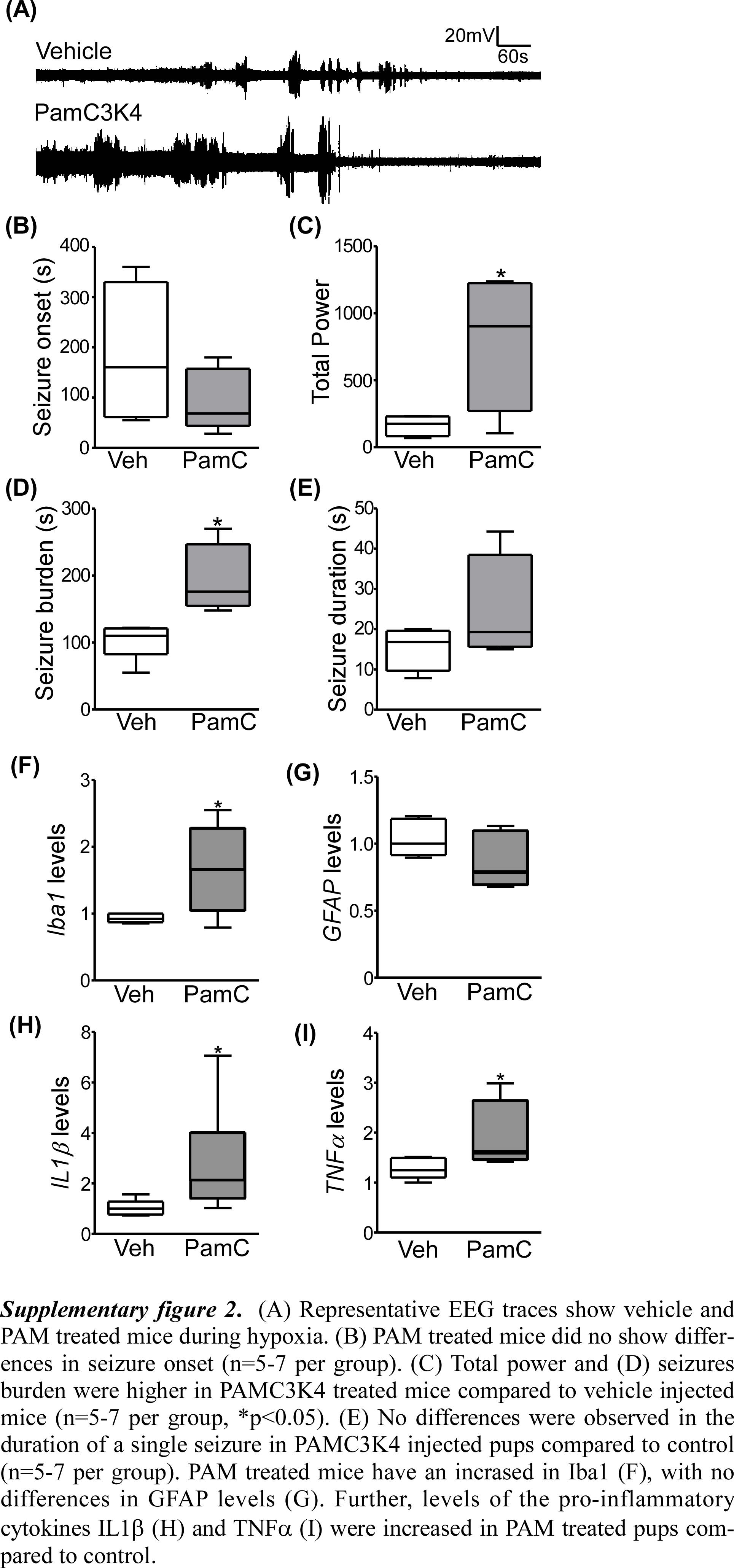

Supplement: Supplementary file 4 [file Image_2.JPEG]
